# Role of Nuclear Lamin A/C in the Regulation of Nav1.5 Channel and Microtubules: Lesson From the Pathogenic Lamin A/C Variant Q517X

**DOI:** 10.3389/fcell.2022.918760

**Published:** 2022-06-29

**Authors:** Roberta De Zio, Giusy Pietrafesa, Serena Milano, Giuseppe Procino, Manuela Bramerio, Martino Pepe, Cinzia Forleo, Stefano Favale, Maria Svelto, Andrea Gerbino, Monica Carmosino

**Affiliations:** ^1^ Department of Biosciences, Biotechnologies and Biopharmaceutics, University of Bari, Bari, Italy; ^2^ Department of Sciences, University of Basilicata, Potenza, Italy; ^3^ ASST Grande Ospedale Metropolitano Niguarda Pathological Anatomy Center, Milano, Italy; ^4^ Department of Emergency and Organ Transplantation, Cardiology Unit, University of Bari Aldo Moro, Bari, Italy

**Keywords:** lamin A/C, arrhythmias, sodium channel, tubulin, electrophysiology

## Abstract

In this work, we studied an *lmna* nonsense mutation encoding for the C-terminally truncated Lamin A/C (LMNA) variant Q517X, which was described in patients affected by a severe arrhythmogenic cardiomyopathy with history of sudden death. We found that LMNA Q517X stably expressed in HL-1 cardiomyocytes abnormally aggregates at the nuclear envelope and within the nucleoplasm. Whole-cell patch clamp experiments showed that LMNA Q517X-expressing cardiomyocytes generated action potentials with reduced amplitude, overshoot, upstroke velocity and diastolic potential compared with LMNA WT-expressing cardiomyocytes. Moreover, the unique features of these cardiomyocytes were 1) hyper-polymerized tubulin network, 2) upregulated acetylated α-tubulin, and 3) cell surface Nav1.5 downregulation. These findings pointed the light on the role of tubulin and Nav1.5 channel in the abnormal electrical properties of LMNA Q517X-expressing cardiomyocytes. When expressed in HEK293 with Nav1.5 and its β1 subunit, LMNA Q517X reduced the peak Na^+^ current (I_Na_) up to 63% with a shift toward positive potentials in the activation curve of the channel. Of note, both AP properties in cardiomyocytes and Nav1.5 kinetics in HEK293 cells were rescued in LMNA Q517X-expressing cells upon treatment with colchicine, an FDA-approved inhibitor of tubulin assembly. In conclusion, LMNA Q517X expression is associated with hyper-polymerization and hyper-acetylation of tubulin network with concomitant downregulation of Nav1.5 cell expression and activity, thus revealing 1) new mechanisms by which LMNA may regulate channels at the cell surface in cardiomyocytes and 2) new pathomechanisms and therapeutic targets in cardiac laminopathies.

## Introduction

Lamin A/C (LMNA) are type-V intermediate filament proteins expressed by the majority of differentiated somatic cells. Both proteins are encoded by the same gene on chromosome 1q22 through alternative splicing events and targeted to the nucleus where they polymerize to form the nuclear lamina, which is a scaffold that underlies the inner nuclear membrane. This mesh of proteins plays multifunctional roles in cell biology. Nuclear lamins are pivotal for the maintenance of cellular and nuclear integrity and for correct intranuclear mechanotransduction, spatial organization of chromatin, regulation of signaling, and gene expression (Carmosino et al., 2014; Gerbino et al., 2018).

Hundreds of different mutations in the *lmna* gene segregate with largely autosomal-dominant conditions identified as laminopathies. Mostly, these diseases affect specifically the striated muscle with a recurrent involvement of the heart. Notably, almost half of the LMNA cardiomyopathy patients succumb to sudden cardiac death as a result of a fatal arrhythmia, and conduction defects associated with LMNA mutations can substantially precede the onset of structural heart modification, meaning that subtle but fatal arrhythmias may occur before any noticeable change in the function (Hasselberg et al., 2018).

Several hypotheses have been postulated to underlying the electrical abnormalities in the heart of laminopathy patients.

We demonstrated that either nuclear fragility or ER stress may increase the rate of apoptosis in cells expressing pathogenic LMNA variants (Forleo et al., 2015; Carmosino et al., 2016). Recently, we also found proinflammatory cytokines deregulation in different LMNA mutant carriers with arrhythmogenic cardiomyopathies (Gerbino et al., 2021). Of note, either apoptosis or inflammation may in turn induce deposition of fibrotic tissue acting as an arrhythmic substrate.

Moreover, the expression and the function of the connexin 43 have been found altered in cardiomyocyte *syncytia* (Gerbino et al., 2017; Borin et al., 2020) and mouse heart (Macquart et al., 2019) expressing pathogenic variants of LMNA, thus accounting for conduction defects associated with those variants.

Electrical disturbance in the heart may also result from the defective electrical impulse generation due to ion channels remodeling of atrial and ventricular myocytes.

Some LMNA variants have been associated with changes in sodium currents. Olaopa and collaborators showed that variants R545H and A287Lfs*193 reduced the peak Na^+^ current when coexpressed with Nav1.5 channel in HEK293 cells (Olaopa et al., 2018). The LMNA V445E mutant also reduced the peak current of I_Na+_ when coexpressed with Nav1.5 channel in HEK293 cells (Liu et al., 2016). Similarly, the expression of LMNA R399C mutant decreases the density of cardiac sodium current in Nav1.5-expressing HEK293 cells (Han et al., 2019). Recently, Savarini et al. showed that iPSC-derived cardiomyocytes expressing LMNA K219T pathogenic variant have altered action potentials, reduced peak sodium current, and diminished conduction velocity (Salvarani et al., 2019).

On the other hand, both peak and late I_Na+_ were significantly increased in cardiomyocytes from Lmna^N195K/N195K^ transgenic mice (Markandeya et al., 2016) with concomitant prolongation of Action Potential (AP) duration.

Of note, the mechanisms through which LMNA mutants may affect Nav1.5 trafficking and activity have not been always elucidated. Interestingly, an epigenetic effect of LMNA K219T variant acting through the *scn5a* gene silencing has been reported (Salvarani et al., 2019).

Overall, regardless of which theory better explains the etiology of cardiac arrhythmia in laminopathies, dissecting the pathogenic mechanisms at the cellular level would offer information on the mechanism by which this nuclear protein may regulate the electrical properties of cardiomyocytes and personalized therapeutic approaches in the field of laminopathies.

Here, we characterized a truncated LMNA variant, Q517X, a pathogenic LMNA variant shown to be associated with dilated cardiomyopathy with conduction abnormalities and neuromuscular disorders (Stallmeyer et al., 2012). In the family involved in our study, however, LMNA Q517X segregates with a severe cardiac phenotype and history of sudden death without any neuromuscular involvement, most likely because of the genetic background of the mutation carriers and environmental influences. The LMNA haploinsufficiency due to the non-sense-mediated decay of the messengers encoding for truncated LMNA variants has been proposed as one of the pathogenic mechanisms in carriers of *lmna* nonsense gene mutations. However, we and others demonstrated the expression of the truncated LMNA variant in the tissues of the carriers (Arbustini et al., 2002; Carmosino et al., 2016). Accordingly, several studies performed on genotype–phenotype correlations in cardiac laminopathy demonstrated that truncation mutations are associated with more severe phenotypes and poor prognosis in LMNA mutation carriers because of the early onset of conduction disturbance, atrial fibrillation, malignant ventricular arrhythmias, and sudden death (van Rijsingen et al., 2012; Nishiuchi et al., 2017). Thus, unmasking the pathogenic mechanisms associated with the expression of C-terminal truncated LMNA variants in cardiomyocytes has high clinical relevance.

At the onset of the cardiomyopathy LMNA Q517X carriers presented sinus node dysfunction, first-, second-, and third-degree atrioventricular block and persistent atrial fibrillation. Years later DCM developed with ventricular arrhythmias and heart failure finally occurred. Since the key pathogenetic mechanism in this cardiomyopathy seems to be the electrical disturbance in the atria, we analyzed the possible effect of LMNA Q517X expression in HL-1 cardiomyocytes. These cardiomyocytes possess a mixed phenotype between an atrial cardiomyocyte and a pacemaker cell, as evident by the ion channels expressed at the plasma membrane of these cells (Claycomb et al., 1998; Yang and Murray, 2011). Consequently, these cells can beat spontaneously in culture for the presence of I_f_ current, but the AP generation is due to Na^+^ inward current through Nav1.5 channel as in adult atrial cardiomyocytes (Strege et al., 2012), thus providing a unique model to investigate the effect of this LMNA mutant in atrial automaticity.

## Materials and Methods

### Patients

The heterozygous nucleotide substitution c.1549C > T in exon 9 of the *lmna* gene, introducing a premature stop codon (*p*.Q517X), was detected in a 36-year-old man referred to our Cardiomyopathy Unit, Cardiology Unit, Department of Emergency and Organ Transplantation, University of Bari Aldo Moro, Bari (Italy). He presented conduction system disorders, premature ventricular complexes, and left ventricle dilatation. Clinical evaluations and molecular analysis were proposed in all first-degree relatives of our index patient. The participants underwent a clinical workup including medical history, physical examination, 12-lead electrocardiogram (ECG), transthoracic echocardiography, and 24-h ECG monitoring. Written informed consent was provided by all participating subjects and was obtained by parents of the minor included in the study. This project was consistent with the principles of the Declaration of Helsinki and was approved by the Ethics Committee of the University Hospital Consortium, Policlinico of Bari, Italy.

### Cell Culture

HL1 cardiomyocytes were cultured in Claycomb Medium (51800C, Sigma-Aldrich) supplemented with 10% fetal bovine serum (F2442, Sigma-Aldrich), Penicillin/Streptomycin 100 U/mL: 100 μg/ml (P4333, Sigma-Aldrich), 2 mM L-Glutamine (G7513, Sigma-Aldrich), and 0.1 mM Norepinephrine [(±)-Arterenol] (A0937, Sigma-Aldrich) in a humidified 5% CO2, 95% O2 incubator at 37°C.

HEK293 cells and HEK293T packaging cells were cultured in Gibco™ DMEM, high glucose, GlutaMAX™ (31966-021, Life Technologies™) supplemented with 10% Gibco™ Fetal Bovine Serum (10270-106, Life Technologies™) and 1% Penicillin-Streptomycin (10,000 U/mL,15140122, Gibco™, Life Technologies™) in a humidified 5% CO_2_, 95% O_2_ incubator at 37 °C.

Cell concentration and viability were assessed using Trypan Blue Stain, 0.4% with LUNA-II™ Automated Cell Counter (Logos Biosystems).

### Generation of LMNA-Expressing HL-1 Stable Clones

HL-1 cells stably expressing LMNA WT or LMNA Q517X were obtained using Lentiviral transduction.

For viral particles production HEK293T packaging cells were plated at 30% confluence on 60‐mm Petri dishes coated with Poly-L-lysine hydrobromide (2,636, Sigma‐Aldrich). After 24 h, cells were co-transfected, using Invitrogen™ Lipofectamine™ 2000 Transfection Reagent (Invitrogen Corporation), with the plasmid encoding the protein of interest (either Lamin WT mCherry-tagged pLV [Exp]-Neo-CMV > mCherry (ns):hLMNA [NM_170707.4] or LMNA Q517X mCherry-tagged pLV [Exp]-Neo-CMV > mCherry (ns):hLMNA [NM_170707.4]*, Vector Builder, CA), two additional plasmids such as an envelope protein VSV-G-expressing plasmid pMD2G and a packaging plasmid pSPAX2-expressing Gag-pol and Tat viral proteins (Addgene) in OPTIMEM (Gibco™ Opti‐MEM™). The day after the transfection the medium was replaced by 3 ml of fresh supplemented DMEM with penicillin–streptomycin. 24-h after, the virus-containing medium was harvested, clarified with 0.45-µm filter, and stored at −80°C, and 3 ml of fresh medium was re-added to the cells. After additional 24-h of incubation, the virus-containing medium was harvested again and pooled with that collected the day before.

For viral transduction, the HL-1 cells were plated in a 6-well plate at 30–50% of confluency, and after adhesion, they were incubated with 500 µl of virus-containing medium at 37°C in a humidified 5% CO_2_ incubator for 18h. The cells were then incubated with 400 μg/ml Geneticin (G418, Gibco, Life Technologies) for 1 week to select stable clones.

### Generation of Transfected HEK293 Cells for Patch Clamp Studies

HEK293 cells were transiently co-transfected with plasmids encoding LMNA WT or LMNA Q517X, the voltage sodium channel Nav1.5, and the Nav1.5 accessory β1 subunit using lipofection following the manufacturer’s instructions (Invitrogen Corporation), 24 h before patch clamp experiments.Where described, HEK293 were transiently co-transfected with plasmids encoding LMNA WT or LMNA Q517X and the voltage K^+^ channel KCNH2 (Supplementary Figure S4).

The generation of the LMNA WT mCherry-tagged construct was previously described (Carmosino et al., 2016). The generation of the LMNA Q517X mCherry-tagged construct was performed by the mutagenesis of the LMNA WT mCherry construct using the “Stratagene’s Quik Change II XL site-directed mutagenesis kit” KIT (Agilent Technologies, United States). The mutagenic primers were designed using the Quick Change Primer Design Program available online at www.agilent.com/genomics/qcpd/$. The mutation was verified by sequencing. Nav1.5 GFP-tagged and β_1_ subunit constructs were kindly provided by Paola Imbrici from Department of Pharmacy-Drug Sciences, University of Bari, Italy. GFP-tagged KCNH2 encoding plasmid was previously characterized by us (De Zio et al., 2019).

### Electrophysiological Recordings

Electrophysiological recordings were performed with the Patch Clamp technique in a whole-cell configuration using the Multiclamp 700B (Axon CNS-Molecular Devices, Sunnyvale, CA, United States) amplifier interfaced with the Axon Digidata 1,500 (Axon Instrument-Molecular Devices, Sunnyvale, CA, United States). Currents were sampled at 10 k Hz and low-pass filtered at 5 kHz. AxoScope 10.4 (Molecular Devices, Sunnyvale, CA, United States) and pClamp 10.4 (Molecular Devices, Sunnyvale, CA, United States) were used to acquire and analyze the data. After gigaseal formation and whole‐cell access, pipette capacitance (Cp) and the membrane capacitance (Cm) were compensated adjusting the Cp fast and the Cp slow setting on the MultiClamp 700B. A Rs stable for the entire experiment and lower than 20 MΩ was considered acceptable. All recordings were performed at room temperature (26 °C) on HL-1 cardiomyocytes stably expressing either LMNA Q517X or LMNA WT and on HEK293 cells transiently co-transfected with the GFP-tagged Nav1.5 channel, its associated β1 subunit, and LMNA Q517X or WT-expression plasmids. Where described HEK293 cells were transiently co-transfected with GFP-tagged KCNH2 and LMNA Q517X or WT-expression plasmids. Fluorescent tags were used for selecting the transfected cells. Borosilicate patch pipettes were pulled to obtain tip resistances of 2–4 MΩ with the P-1000 Pipette puller (SUTTER INSTRUMENT, Novato, CA 94949, United States).

For the electrophysiological recordings on HL-1 cardiomyocytes, cells stably expressing either LMNA WT or LMNA Q517X were plated at low confluence, on 35‐mm Petri dishes coated with gelatin-fibronectin (5 μg/ml Fibronectin, F1141, Sigma-Aldrich, in 0.02% Gelatin from bovine skin, G9391, Sigma-Aldrich), the day before the experiments. After complete cell adhesion, 10-μM Colchicine (C-9754 Sigma) was added to the complete Claycomb media for an overnight treatment in a humidified 5% CO_2_, 95% O_2_ incubator at 37 °C, when required. HL-1 spontaneous action potentials were recorded using an extracellular solution contained (in mM): 138 NaCl, 4 KCl, 1 MgCl_2_, 1,8 CaCl_2_, 10 Hepes, 10 Glucose (pH of 7.4, adjusted with NaOH, and osmolarity of 290 ± 10 mmol/kg, adjusted with mannitol) and an internal pipette solution contained (in mM): 144 KCl, 2 MgCl_2_, 10 Hepes, 5 EGTA (pH of 7.2 adjusted with KOH and osmolarity of 280 ± 10 mmol/kg, adjusted with mannitol). Spontaneous action potentials (APs) were recorded in current clamp mode without current injection and analyzed for the following parameters: amplitude (expressed in mV), overshoot (expressed in mV), upstroke (expressed as ΔV/Δt), maximum diastolic potential (MDP, expressed in mV), and threshold (expressed in mV).

For the electrophysiological recordings on HEK293 cells transiently expressing either LMNA WT or LMNA Q517X, Nav1.5 channel and its β1 subunit were plated at low confluence the day before the experiments on 35‐mm Petri dishes coated with Poly-L-lysine hydrobromide for 20 min at room temperature (2636 Sigma‐Aldrich). After complete adhesion, 10-μM Colchicine (C-9754 Sigma) was added to the complete Glutamax media for an overnight treatment in a humidified 5% CO_2_, 95% O_2_ incubator at 37°C, when needed.

The whole-cell Na^+^ current (I_Na_) in the HEK293 cells was recorded using a bath solution contained (in mM): 140 NaCl, 5 KCl, 1 CaCl_2_, 1 MgCl_2_, 10 HEPES, 5 Glucose (pH of 7.4, adjusted with NaOH, and osmolarity of 290 ± 10 mmol/kg, adjusted with mannitol) and an internal pipette solution contained (in mM): 140 CsCl, 10 NaCl, 10 HEPES, 1 EGTA, pH 7.4, adjusted with CsOH. The I_Na_ current was measured by 20 ms depolarizing voltage steps ranging from −90 to +60 mV in 5 mV increments after a holding potential of −120 mV to remove the possible inactivation.

The peak values of I_Na_ recorded at each voltage step were normalized to the cell capacitance and reported (as current density, in pA/pF) plotted against the membrane potential (Vm). Additionally, a scatter plot was used to show the distribution of the maximum I_Na_ peaks of each experiment (expressed as current density, in pA/pF).

The conductance (G) was determined using the modified Ohm’s law equation:

$$G=I/\left(Vm-V_{rev}\right),$$

where I is the peak current Na^+^ evoked at the membrane potential Vm and V_rev_ the reversal potential for the Na^+^ in our experimental condition. The conductance values were normalized with respect to the maximum conductance value (G/G_max_) and plotted against the voltage. The conductance voltage curves were fitted with the Boltzmann function

$$G_{Na}=G_{Na,\max}/\left\{1+\exp\left[\left(V1/2-Vm\right)/K\right]\right\}$$

to yield the membrane potentials at half-maximal conductance (V1⁄2), and the slope factors (K). Additionally, the scatter plots were used to show the distribution of V1/2 and the K of each experiment for both experimental conditions.

Electrophysiology on KCNH2-expressing HEK293 cells was conducted as previously described (De Zio et al., 2019).

### Laser Confocal Immunofluorescence Analysis

For the immunofluorescence analysis HL-1 cardiomyocytes were plated on 12-mm diameter glass coverslips coated with gelatin-fibronectin (5 μg/ml Fibronectin, F1141 Sigma-Aldrich, in 0.02% Gelatin from bovine skin, G9391 Sigma-Aldrich). After complete cell adhesion, 10 μM Colchicine (C-9754 Sigma) was added to the complete Claycomb media for an overnight treatment in a humidified 5% CO_2_, 95% O_2_ incubator at 37°C, when needed. Cells were subjected to immunofluorescence analysis as previously described (Gerbino et al., 2016). Primary antibodies used were: anti-β-Tubulin antibody produced in mouse (1:1,000 in PBS‐BSA 1%, T5293 Sigma-Aldrich), the anti-mCherry antibody (1:500 in PBS‐BSA 1%, ab167453 Abcam), the anti-γ-tubulin antibody (1:200 in PBS‐BSA 1%, T6557 Sigma-Aldrich). After 3 washes in PBS the cells were incubated with the appropriate Alexafluor-conjugated secondary antibodies (Life Technologies™) for 1 h at RT. The confocal images were obtained with a laser scanning fluorescence microscope Leica TSC-SP2 (HCX PL APO, ×63/1.32–0.60 oil objective); 8-bit images were saved at 1,024 × 256 and acquired using the Leica Confocal Software^®^. Fluorescence was quantified in a selected region of interest (ROI) using Fiji (https://imagej.net/Fiji).

For immunofluorescence analysis on cardiac biopsies, 10-µm-thick paraffine-embedded sections of heart biopsies were deparaffinized by incubation at room temperature with histolemon for 10 min and rehydrated with a graded series of ethanol. Sections were then subjected to antigen retrieval by boiling in citrate buffer (10 mM sodium citrate, pH 6) and incubated with PBS-1% SDS for 10’. After blocking with 1% bovine serum albumin in PBS for 30’, the sections were incubated with anti-LAMIN A/C (1:100 in PBS‐BSA 1%, #2032 Cell Signaling) and then with Alexa Fluor-conjugated secondary antibodies (Life Technologies). The images were acquired as described for HL-1 cells.

### Cell Surface Biotinylation

For biotinylation experiments, HL-1 cardiomyocytes were plated in wells of a 6‐multiwell cell culture support coated with gelatin-fibronectin (5 μg/ml Fibronectin, F1141, Sigma-Aldrich, in 0.02% Gelatin from bovine skin, G9391 Sigma-Aldrich), at a density to be 100% at the time of biotinylation. When necessary cells were incubated with 10 μM colchicine overnight before biotinylation. The procedure was previously described with some modification (Carmosino et al., 2008). Briefly, the HL-1 cells were washed twice in cold phosphate buffer saline (PBS, pH 8) containing 1 mM Ca^2+^ and 0.5 mM Mg^2+^ (PBS-CM) and incubated for 30 min at 4 °C in 2 mg/ml Biotin 3-sulfo-N-hydroxysuccinimide ester sodium salt (B5161 Sigma-Aldrich) in PBS-CM. After three washes in PBS-CM the exceeding biotin was removed washing the cells twice, 10 min at 4 °C, with a quenching solution (50 mM NH_4_ Cl in PBS). After biotin binding cells were lysed in Lysis buffer (in mM: 20 Tris-HCl pH 8, 150 NaCl, 5 EGTA, and 1% triton X-100) supplemented with 1X protease inhibitor mixture (Roche), and phosphatase inhibitor (in mM: 10 NaF, 100 orthovanadate, 15 pyrophosphate), for 30 min on ice and sonicated at 60 amplitude with Vibra‐cell^®^ (Sonics and Materials Inc.) 3 times for 15 s. After sonication, membranes were pelleted at 13000 rpm for 30 min at 4 °C and the supernatant was collected. Protein concentrations were determined by Bradford protein assay. An equal amount of total protein from each group was incubated with streptavidin-agarose beads (69203 Novogen) overnight at 4 °C in a rotating device. Beads were then washed with Lysis buffer supplemented with protease and phosphatase inhibitors, and proteins were eluted by incubation with Laemmli buffer and freshly added 100 mM DTT, for 20 min at 95 °C and 1,000 rpm. The protein samples were subjected to SDS-Page and western blot analysis as described below.

### Cell and Tissue Lysates

HL-1 cells, 100% confluent, plated in the wells of a 6‐multiwell cell culture support coated with gelatin-fibronectin (5 μg/ml Fibronectin, F1141 Sigma-Aldrich, in 0.02% Gelatin from bovine skin, G9391 Sigma-Aldrich) were placed on ice, washed in PBS, and exposed to RIPA buffer (in mM: 150 NaCl, 10 Tris/HCl, 1% Triton X‐100, 0.1% SDS, 1% deoxycholate‐Na, 5 EDTA; pH 7.2) supplemented with protease and phosphatase inhibitors (in mM: 10 NaF, 100 orthovanadate, 15 pyrophosphate). Cells were then sonicated at 60 amplitude with Vibra‐cell^®^ (Sonics and Materials Inc.) and membranes pelleted at 13000 rpm for 30 min at 4°C. Protein concentrations were determined by Bradford protein assay. Proteins were de‐natured in 1 × Laemmli Sample Buffer (Bio‐Rad), 50 mM DTT and subjected to SDS-PAGE and western blotting as described below.

Lysates were also prepared from cardiac biopsies from the index patient’ transplanted heart. These biopsies were kindly provided by Niguarda Pathological Anatomy Center, Milano, Italy, where the index patient underwent heart transplantation. Twenty µm-thick paraffine-embedded sections of heart biopsies were deparaffinized by incubation at room temperature with histolemon for 10 min and rehydrated with a graded series of ethanol. The sections were then pelleted at 16000 × g for 5 min and homogenized in 500 mM Tris-HCl pH 8 and 2% SDS at 90°C for 3 h. The samples were centrifugated for 30 min at 16000 × g at 4°C and the supernatants were subjected to SDS-PAGE and western blotting as described below. Lysates from an explanted heart of a patient with heart disease not related to LMNA mutations were used as control, as previously described (Carmosino et al., 2016).

### Western Blot

Protein samples were electrophoresed on 7.5% or 10% polyacrylamide SDS gel (Mini-PROTEAN TGX Stain-Free Precast Gels; Bio‐Rad) and transferred on 0.2-µm PVDF membrane (Trans-Blot Turbo Mini 0.2 µm PVDF Transfer Packs #1704156; Bio‐Rad) using the Trans-Blot Turbo Transfer System (Bio‐Rad). After blocking at room temperature for 1 h in TBST-5% milk or in TBST‐BSA 3% (TBST in mM: 50 Tris, 150 NaCl, 0.1% Tween‐20; pH 7.4), blots were incubated overnight at 4°C with the following antibodies in blocking buffer: anti-Lamin A/C (1:1000 in TBST‐1% BSA; 2032 Cell Signaling); anti-mCherry antibody (1:1000 in TBST‐BSA 1%; ab167453 Abcam); anti-Nav1.5 (1:700 in TBST‐1% BSA; S8809 Sigma‐Aldrich), anti-acetylated α-tubulin antibody (1:1000 in TBST‐1% BSA; T7451 Sigma‐Aldrich), anti-β-tubulin antibody (1:1000 in TBST‐1% BSA; T5293 Sigma‐Aldrich), anti-AKT (1:2000 in TBST‐1% BSA; 4691 Cell Signaling), anti-phospho-AKT (Ser473) XP^®^ (1:4000 in TBST‐1% BSA; 4060 Cell Signaling), anti-ERK1/2 p44/42 MAPK (ERK1/2) (1:1000 in Milk‐5% BSA; 4695 Cell signaling), anti-phospho ERK1/2 p44/42 MAPK (ERK1/2) (1:2000 in Milk‐5% BSA; 4695 Cell signaling); the anti-mCherry antibody (1:500 in PBS‐BSA 1%, ab167453 Abcam. As secondary antibodies were used the goat anti-mouse IgG‐HPR conjugate (1:5000 in TBST‐3% BSA; Bio‐Rad) and an anti‐rabbit IgG‐peroxidase antibody produced in goat (1:5000 in TBST‐ BSA 3%; Sigma‐Aldrich) depending on the primary antibodies’ species. The immunoreactive bands were detected with a ChemiDoc™ System (Bio‐Rad). Densitometric analysis was performed by Image Lab 6.0 software. For protein load controls, the stain‐free technology from Bio‐Rad was used.

### Statistical Analysis

GraphPad Prism 6 was used for the statistical analysis and graph representation of the data. Data are given as mean ± standard error of the mean. Statistical analysis was performed using one- or two‐way ANOVA test or with Student’s t-test for unpaired data depending on the data set analyzed.

## Results

### Clinical Findings

**FIGURE 1:**
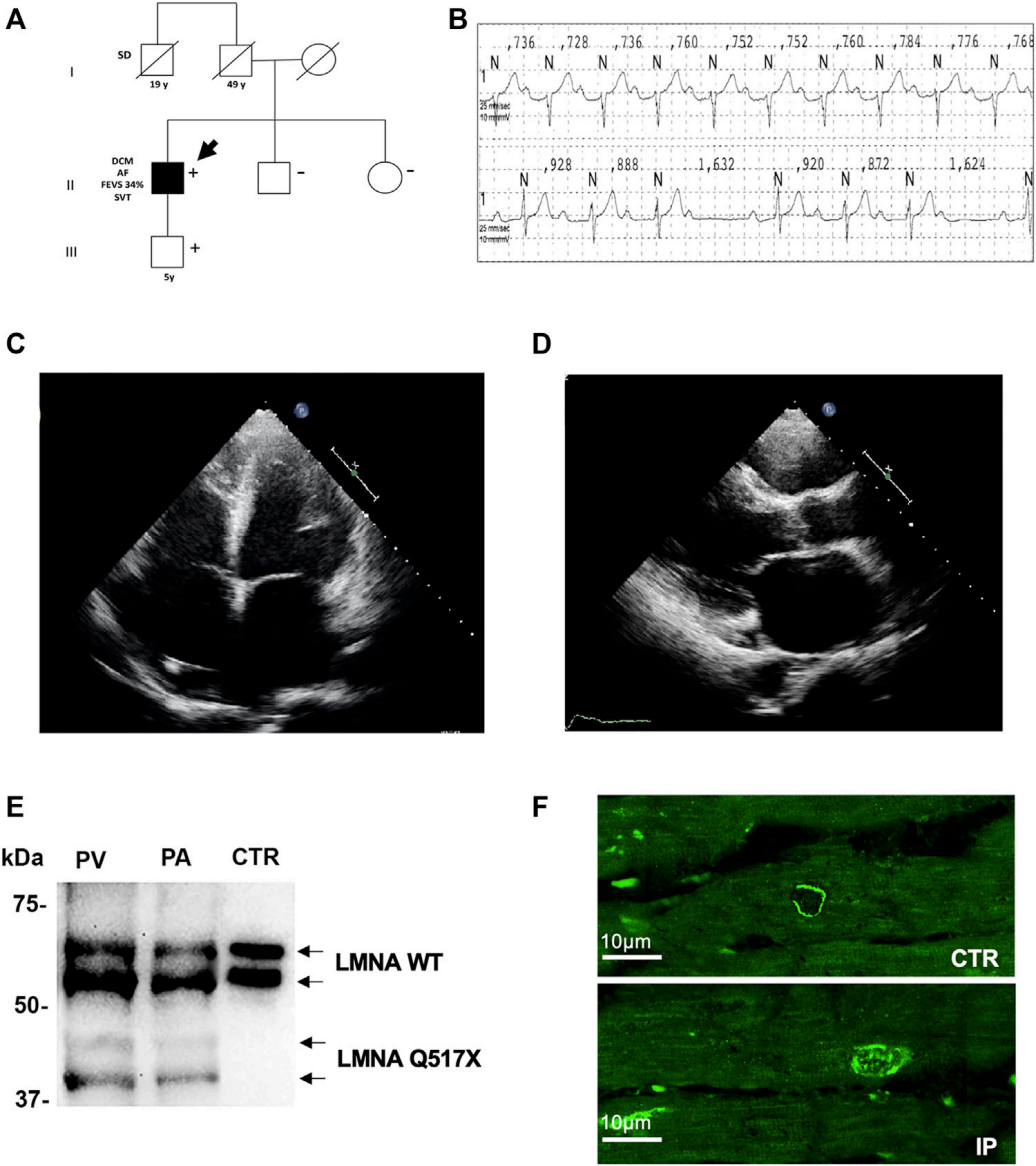
**(A)** Pedigree of the family carrying the LMNA Q517X pathogenic variant. The filled symbols indicate clinically affected individuals, the diagonal slashes indicate deceased individuals; +/− indicate positive/negative for the mutation; the arrow indicates the index patient. **(B)** ECG data from index patient showing sinus rhythm, first-degree atrioventricular block (upper trace), and type 1 second-degree atrioventricular block (lower trace) recorded during a 24-h ECG monitoring. **(C)** Apical 4C views of echocardiographic imaging from index patient showing a spherical, severely dilated, and markedly hypocontractile left ventricle. **(D)** In the PLAX view of echocardiographic imaging from index patient, showing a highly dilated left atrium. **(E)** Western blot analysis of whole lysate of left atrium (PA) and (PV) ventricular myocardial tissue from the mutant carrier (black arrow in A) and control (CTR) heart biopsies. **(F)** Representative confocal immunofluorescence images of LMNA in heart biopsies from a control patient (CTR) and from the index patient (IP). ECG, electrocardiogram; PLAX view, parasternal long-axis view.

The heterozygous nucleotide substitution c.1549C > T in exon 9 of the *lmna* gene introduced a premature stop codon producing a truncated version of the Lamin A/C (LMNA) protein after amino acid 517 (Q517X). This mutation was detected in a 36-year-old man referred to our Cardiomyopathy Unit, Cardiology Unit, Department of Emergency and Organ Transplantation, University of Bari Aldo Moro, Bari (Italy). The index patient had a family history of sudden death (father’s brother died suddenly at the age of 19 during his sleep). The family members available for clinical evaluations and molecular analysis were the index patient’s son and sister. The 5-year-old son carried the LMNA Q517X mutant and was asymptomatic, free of arrhythmias, and showed normal cardiac function, thus suggesting incomplete and age-related penetrance of the mutation. The 43-year-old sister was LMNA mutation-negative, clinically asymptomatic, and had no arrhythmias or structural cardiac abnormalities (Figure 1A, 5 years). The index patient (the arrow in the family three) presented with conduction system disturbances such as sinus node dysfunction, first-, second-, and third-degree atrioventricular block (Figure 1B), numerous repetitive premature ventricular complexes (PVCs), recurrence of persistent atrial fibrillation (Supplementary Figure S1A), and biatrial and biventricular dilation. Based on the electrocardiographic and echocardiographic findings, in combination with a positive family history of sudden death, he received a dual chamber implantable cardioverter-defibrillator (ICD) in primary prevention. Years later, 24-h Holter and ICD remote monitoring showed ventricular fibrillation (VF) discontinued by appropriate ICD shocks, after ineffective anti-tachycardia pacing (Supplementary Figure S1B). Moreover, echocardiogram showed spherical, severely dilated (LVEDVi 92 ml/m2 and LVESVi 69 ml/m2) and markedly hypocontractile (LVEF 25%) left ventricle (Figure 1C) as well as highly dilated left atrium (Figure 1D). At the age of 49, the patient underwent heart transplant for both sustained VT/VF recurrences as a storm and severe impairment of left ventricular function. Western blot experiments on samples of ventricular and atrial myocardium obtained from the index patient at the time of cardiac transplantation confirmed the expression of the truncated LMNA variant in index patient’s heart. As control, we used cardiac muscle tissues from a patient who experienced heart transplantation due to an ischemic heart disease not related to LMNA mutations (Figure 1E). The immunofluorescence confocal analysis using anti-LMNA antibodies on heart biopsies showed LMNA results expressed in protein aggregates in the nuclei of LMNA Q517X carrier’s heart (Figure 1F, IP).

### LMNA Q517X Alters Action Potential Properties in HL-1 Cardiomyocytes

**FIGURE 2:**
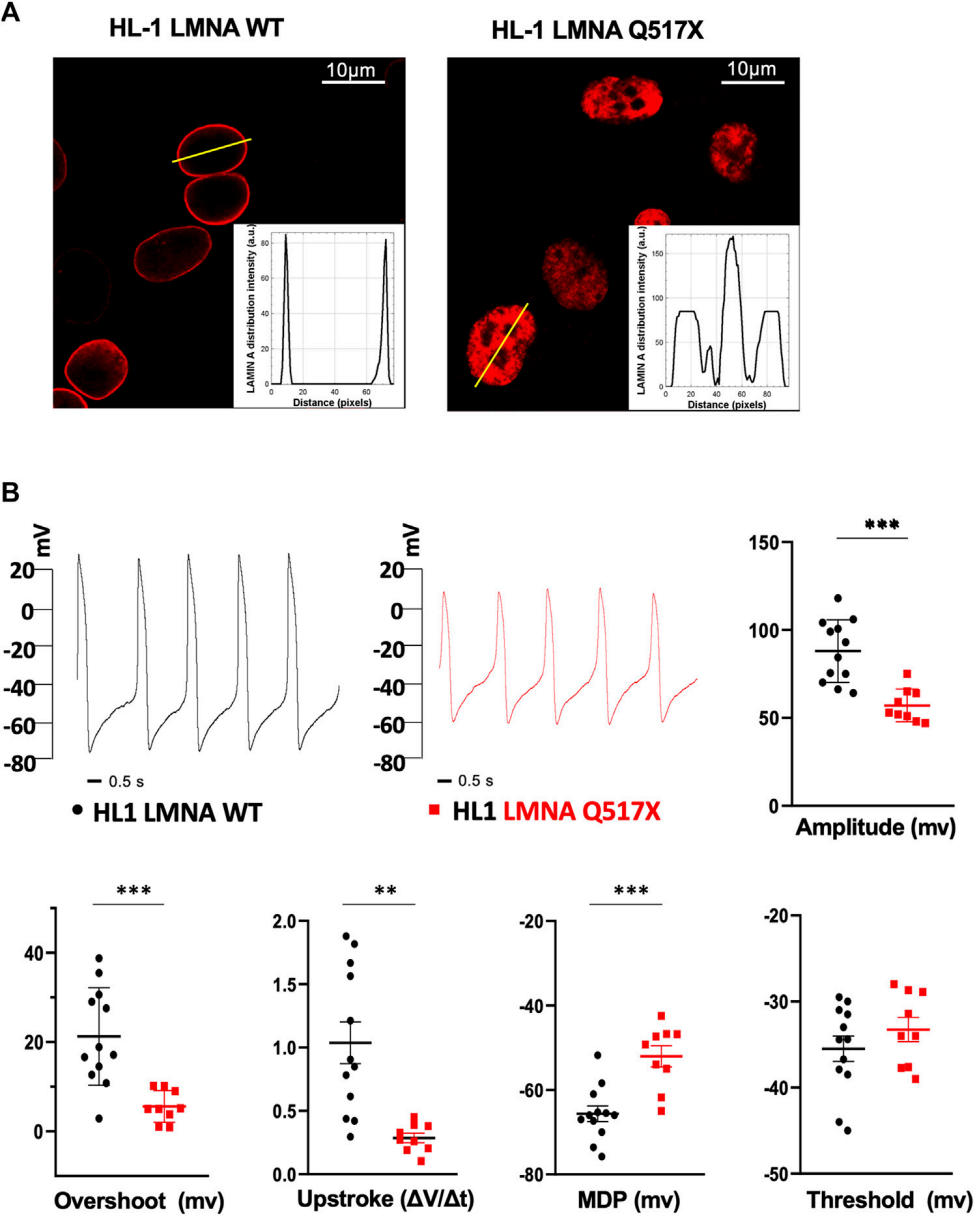
**(A)** Representative confocal immunofluorescence images of LMNA WT and Q517X in stably transfected HL-1 cardiomyocytes. The insets report the fluorescence distribution along the line (yellow line) crossing nuclei in each cell line. **(B)** Representative AP recordings by whole-cell patch clamp in the current clamp configuration in LMNA WT (black trace) and LMNA Q517X-expressing HL-1 cells (red trace) and scatter plots for the analysis of AP parameters in LMNA WT (black dots, N = 12) and LMNA Q517X-expressing HL-1 cells (red squares, N = 9); ***p < 0.001; **p < 0.01; Student’s t-test for unpaired data.

When stably expressed in HL-1 cardiomyocytes, LMNA Q517X abnormally aggregated at the nuclear envelope and accumulated within the nucleoplasm as clearly shown by the fluorescence distribution profile (Figure 2A, HL-1 LMNA WT, HL-1 LMNA Q517X, insets). This peculiar nuclear distribution is consistent with that of LMNA observed *in vivo* in the index patient’s heart (Figure 1E).

To evaluate the effect of LMNA Q517X expression on the membrane electrical properties in cardiomyocytes, we analyzed the biophysical properties of spontaneous action potentials in HL-1 cardiomyocytes expressing either LMNA WT (Figure 2B, HL-1 LMNA WT, black trace) or Q517X (Figure 2B, HL-1 LMNA Q517X, red trace) by whole-cell patch clamp recordings in current clamp. The shape of the spontaneous action potential generated by cardiomyocytes expressing the LMNA mutant variant exhibited significant reduction in Action Potentials (APs) amplitude (Figure 2B, Amplitude in mV: black dots 88.04 ± 5.13 vs red squares 57.11 ± 3.11), APs overshoots (Figure 2B, Overshoot in mV: black dots 21.25 ± 3.14 vs red squares 5.58 ± 1.17), maximum upstroke velocity (Figure 2B, Upstroke in ΔV/Δt: black dots 1.03 ± 0.16 vs red squares 0.28 ± 0.03), and maximum diastolic potential (MDP) (Figure 2B, MDP in mV: black dots 65.63 ± 1.86 vs red squares −52.03 ± 2.50) when compared to control cells; while no significant changes were detected in the APs’ threshold between the two experimental conditions (Figure 2B, threshold in mV: black dots −35.49 ± 1.48 *vs* red squares −33.25 ± 1.41).

### LMNA Q517X Alters Tubulin State and Decreased the Expression of Nav1.5 at the Plasma Membrane in HL-1 Cardiomyocytes

**FIGURE 3:**
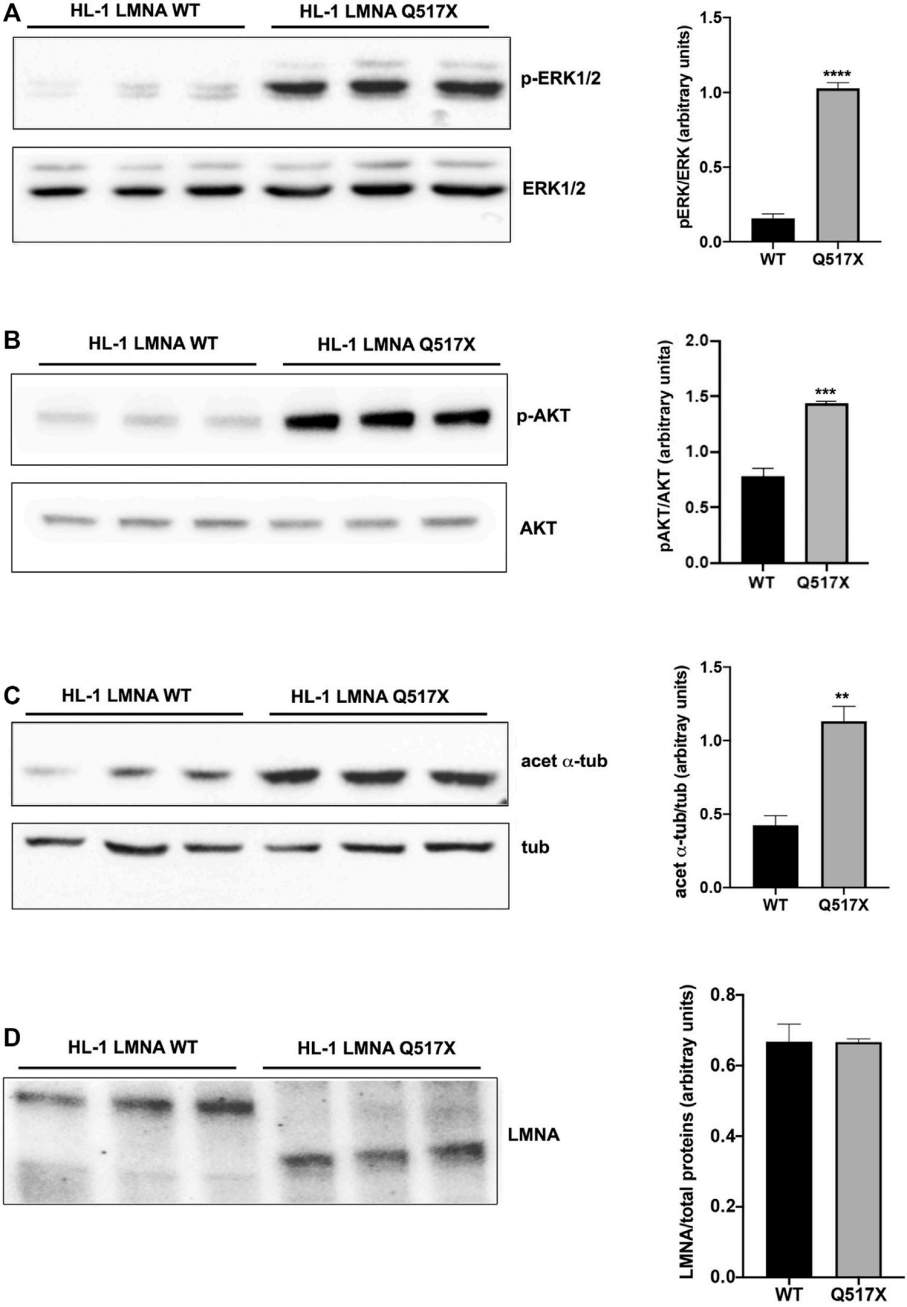
**(A)** Left panel: representative western blot of p-ERK1/2 and ERK1/2 in lysates from LMNA WT and LMNA Q517X-expressing HL-1 cells; right panel: normalized densitometric analysis of p-ERK1/2 immunoreactive bands in LMNA WT and Q517X-expressing HL-1 cells. **(B)** Left panel: representative western blot of p-AKT and AKT in lysates from LMNA WT and LMNA Q517X-expressing HL-1 cells; right panel: normalized densitometric analysis of p-AKT immunoreactive bands in LMNA WT and Q517X-expressing HL-1 cells. **(C)** Left panel: representative western blot of acetylated α-tubulin (acet α-tub) and α-tubulin (tub) in lysates from LMNA WT and LMNA Q517X-expressing HL-1 cells; right panel: normalized densitometric analysis of acetylated α-tubulin immunoreactive bands in LMNA WT and Q517X-expressing HL-1 cells. **(D)** Left panel: representative western blot of mCherry-tagged LMNA in lysates from LMNA WT and LMNA Q517X-expressing HL-1 cells; right panel: normalized densitometric analysis of m-Cherry tagged LMNA immunoreactive bands. The data are means of 3 independent experiments. ****p < 0.0001 ***p< =0.001; **p < 0.01; Student’s t test for unpaired data.

**FIGURE 4:**
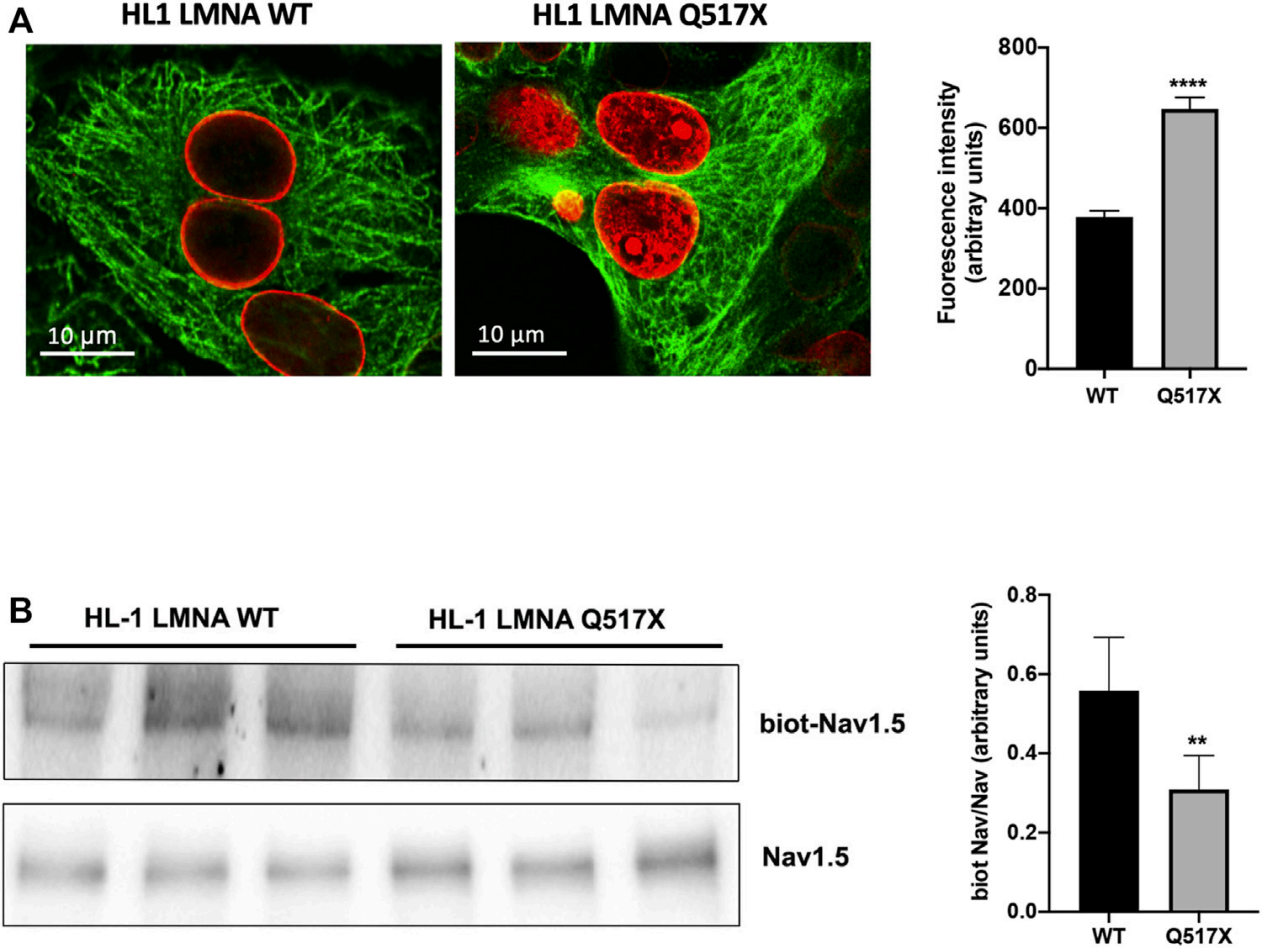
**(A)** Left panel: representative confocal laser immunofluorescence images in LMNA WT and LMNA Q517X-expressing HL-1 cells (in red LMNA, in green β-tubulin); right panel: region of interest (ROI) for tubulin fluorescence quantification in LMNA WT and Q517X-expressing HL-1 cells. N = 15 for each experimental condition. **(B)** Left panel: representative western blots of Nav1.5 α subunit in cell surface biotinylated proteins (biot-Nav1.5) and in cell lysates (Nav1.5) from LMNA WT and Q517X-expressing HL-1 cells; right panel: normalized densitometric analysis of biot-Nav 1.5 immunoreactive bands in LMNA WT and Q517X-expressing HL-1 cells. The data are means of 3 independent experiments. ****p < 0.0001; **p < 0.01; Student’s t test for unpaired data.

In LMNA WT and Q517X-expressing cardiomyocytes we studied the profile of key signaling molecules, already found to be dysregulated in lamin cardiomyopathies. Specifically, enhanced phosphorylation of ERK1/2 and AKT and decreased α-tubulin acetylation have been reported in the heart of Lmna ^H222P/H222P^ mice (Muchir et al., 2007; Choi et al., 2012). Here, we found that the activation profiles of ERK1/2 and AKT, quantified as the expression of their phosphorylated forms, were significantly increased in Q517X cardiomyocytes when compared with their control in accordance with the previous findings (Figures 3A,B). However, acetylated α-tubulin significantly increased in Q517X cardiomyocytes as unique feature of this mutant variant (Figure 3C). The expression of LMNA variants is comparable in the HL-1 clones used in the study (Figure 3D). It has been accepted that acetyl transferase has major affinity for polymerized tubulin (Janke and Chloë Bulinski, 2011); thus, we looked at the polymerization state of tubulin meshwork by fluorescence confocal analysis and found that it underwent hyper-polymerization in Q517X-expressing cardiomyocytes compared with their controls (Figure 4A). Quantitative analysis of fluorescence intensity showed that tubulin density significantly increased by approximately 50% in LMNA Q517X-expressing cardiomyocytes compared with their controls (Figure 4A, right panel). The perinuclear positioning of microtubule-organizing center (MTOC) was not affected by the expression of LMNA Q517X variant in these cells (Supplementary Figure S2), thus suggesting that the physical connection between the nucleus and microtubules is preserved upon LMNA Q517X expression.

It has been previously reported that the treatment with the anticancer drug Taxol, which polymerizes the cytoskeleton protein tubulin, may evoke cardiac arrhythmias reducing both the Nav1.5 expression at the plasma membrane and the Nav1.5 activation rate (Casini et al., 2010).

We indeed semi-quantified membrane Nav1.5 expression using cell surface biotinylation experiments in cardiomyocytes expressing either LMNA Q517X or WT. We found that the cells surface expression of Nav1.5 was significantly downregulated by approximately 40% in LMNA Q517X-expressing cardiomyocytes at the plasma membrane but not in the total lysate (Figure 4B).

Currents in HL-1 cardiomyocytes have been previously shown to have genotypic, phenotypic, and electrophysiologic properties similar to adult atrial cardiomyocytes, with the upstroke phase of the AP due to I_Na_ through Nav1.5 channel (Strege et al., 2012). Accordingly, we measured a significant decrease in tetrodotoxin-sensitive inward currents in LMNA Q517X-expressing cardiomyocytes (Supplementary Figure S3). Of note, outward currents were not affected by the expression of LMNA Q517X in HL-1 cardiomyocytes (Supplementary Figure S3). Indeed, the altered AP parameters registered in LMNA Q517X-expressing cardiomyocytes (Figure 3B) may be actually explained by a decreased density of Nav1.5 at the plasma membrane in these cells.

### LMNA Q517X Significantly Alters Nav1.5 Function in HEK293 Cells

**FIGURE 5:**
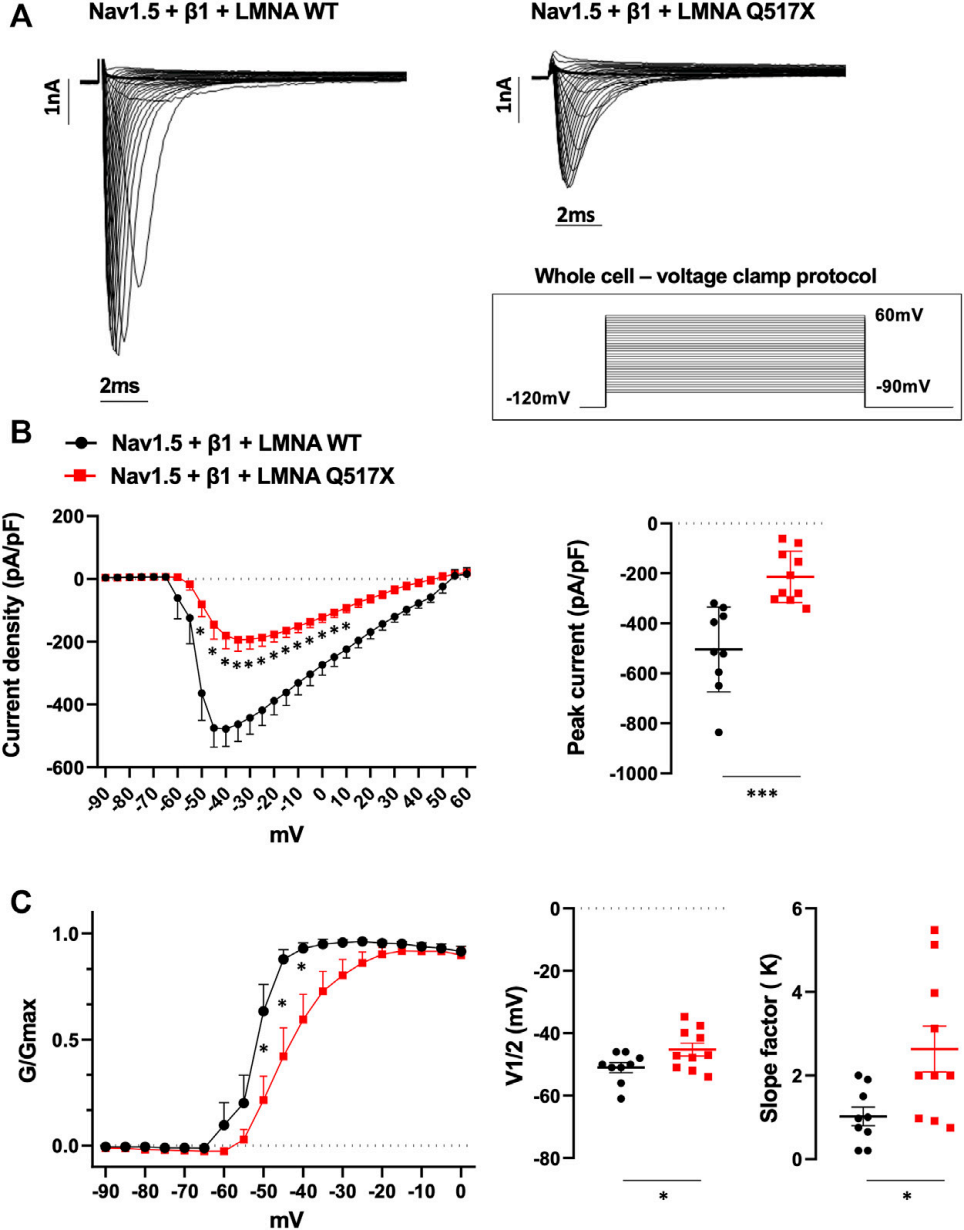
**(A)** Representative traces of inward Na^+^ currents evoked in HEK293 cells expressing either LMNA WT or LMNA Q517X, under 20s depolarizing steps from −90mV to +60 mV (with 5-mV increments) starting from an holding potential of −120 mV. **(B)** Left panel: current density (pA/pF)–voltage relationships (I–V curve) of the Na^+^ currents recorded for LMNA WT (black trace, *N* = 9), and LMNA Q517X-expressing HEK293 cells (red trace, *N* = 10); *p* < 0,0001 from −50 mV to −20 mV; *p*-value< 0.001 at F02D15 mV an −10 mV; *p*-value < 0,01 at −5 mV and *p*-value < 0,05 at 0 mV and 5 mV, two-way Anova. Right panel: scatter plot of the maximum peak sodium currents (pA/pF) for LMNA WT (black dots) and LMNA Q517X-expressing HEK293 cells (red squares); ***p < 0.001, Student’s *t* test for unpaired data). **(C)** Left panel: conductance voltage (G/Gmax) relationships of Na^+^ currents (G–V curve) obtained for LMNA WT (black trace, N = 9) and LMNA Q517X-expressing HEK293 cells (red trace, N = 10); *p*-value < 0,0001 at −50 mV and −45 mV; < 0.001 at −40 mV, two-way Anova. Right panel: scatter plots of the half-maximal conductance (V1⁄2) and the slope factors (K) in LMNA WT (black dots) or LMNA Q517X-expressing HEK293 cells (red squares), respectively; *p < 0.01 Student’s *t* test for unpaired data.

To better analyze Nav1.5 biophysics in the presence of LMNA Q517X we moved in HEK293 cells. Figure 5A shows representative Na^+^ currents measured in HEK293 cells coexpressing Nav1.5, its β1 subunits (Nav1.5 + β1) and either LMNA WT or Q517X. As shown by the current–voltage curve (Figure 5B; black line for Nav1.5 + β1 + LMNA WT, red line for Nav1.5 + β1 + LMNA Q517X), the peak sodium current density evoked by depolarizing steps of currents was significantly decreased in cells expressing LMNA Q517X when compared with control cells expressing LMNA WT. The scatter plot of the maximum peak sodium currents clearly showed an up to 63% decrease in current density upon LMNA Q517X expression (peak current in pA/pF: black dots −504.46 ± 56.72 *vs* red squares −213.59 ± 32.380). This is consistent with the reduced expression of Nav1.5 at the plasma membrane shown above (Figure 4A). The voltage dependency of the steady-state activation for LMNA Q517X was shifted toward positive potentials with respect to WT (Figure 5C). The scatter plot of V1/2 showed a slight but significative shift of approximately 6 mV toward positive values for this parameter upon LMNA Q517X expression (Figure 5C, V1/2: black dots −51 ± 4.8 mV vs red squares −45.25 ± 2.060 mV), thus indicating the need of a more intense depolarization for the activation of Nav1.5 in these cells. The slope factor of activation (K) was significantly increased upon LMNA Q517X expression (Figure 5C, slope factor in mV: black dots 1.019 ± 0.22 vs red squares 2.635 ± 0.546), suggesting an alteration in voltage dependence of Nav1.5 activation in LMNA-Q517X-expressing HEK293 cells.

Interestingly, it has been also proved that a high prevalence of variants in K^+^ channel encoding genes such as KCNH2 might control atrial repolarization and predispose carriers to atrial fibrillation (10.1161/circep.114.002519; 10.1093/eurheartj/ehm619). Indeed, outward K^+^ currents through KCNH2 channel were analyzed in HEK293 cells transiently expressing KCNH2 together with WT or Q517X LMNA. This K^+^ channel is highly expressed in HL-1 cells where it is involved in the repolarization phase of AP (Stimers et al., 2015). We found that the biophysics of this channel was not affected by the expression of LMNA Q517X variant (Supplementary Figure S4).

### Tubulin State Manipulation Recovers AP Parameters in LMNA Q517X-Expressing HL-1 Cardiomyocytes and Nav1.5 Biophysics in HEK293 Cells

Alpha tubulin hyper-polymerization and hyper-acetylation are the unique features of LMNA Q517X-expressing HL-1 cardiomyocytes, likely accounting for the AP abnormalities in these cells.

**FIGURE 6:**
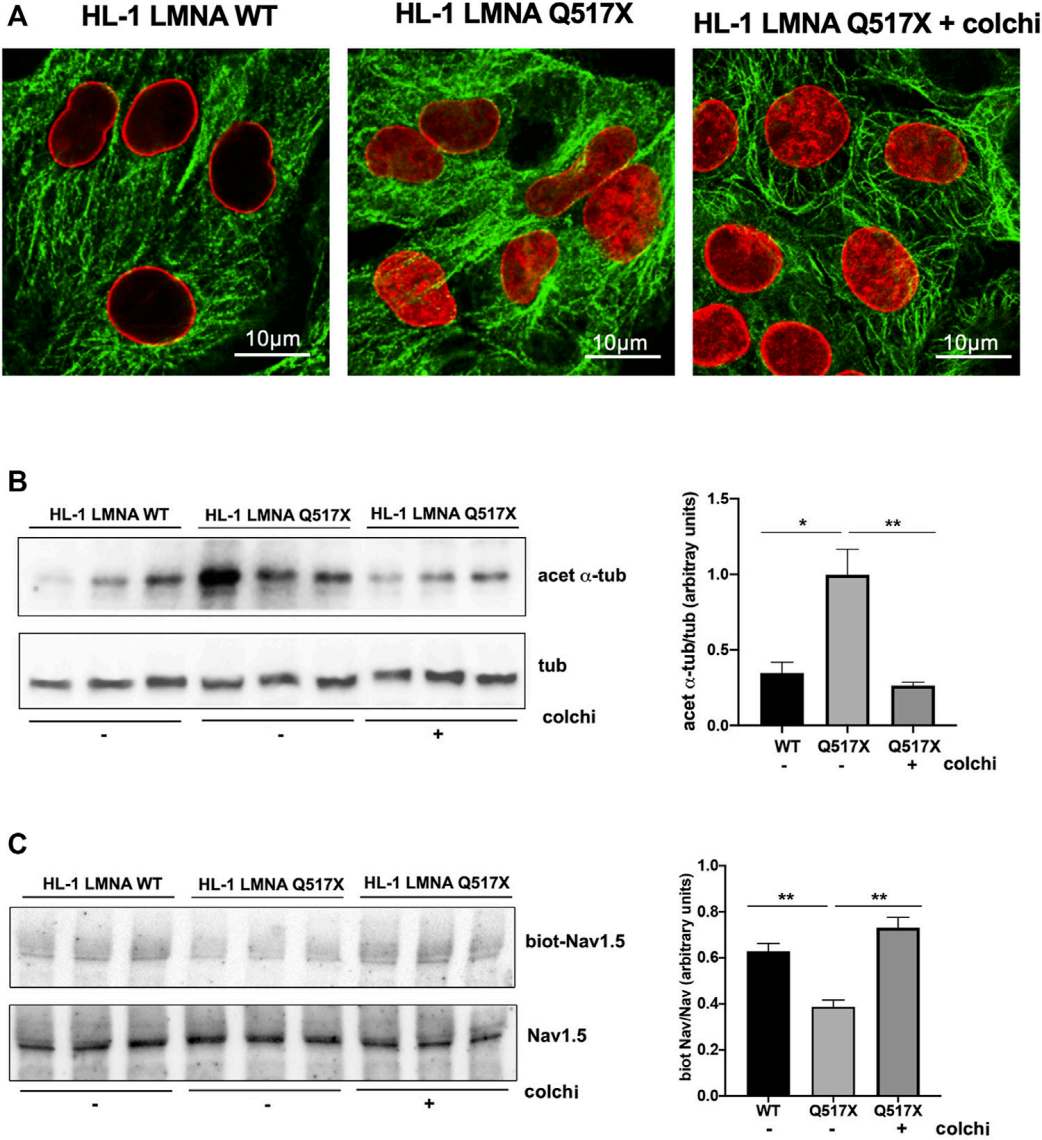
**(A)** Representative immunofluorescence confocal images of HL-1 cells expressing either LMNA WT or LMNA Q517X, the latter treated or not with colchicine (+colchi). **(B)** Left panel: representative western blot of acetylated α tubulin (acet α-tub) and total α tubulin (tub) in the absence (–) or the presence (+) of colchicine (colchi) in LMNA WT and LMNA Q517X-expressing HL-1 cells; right panel: normalized densitometric analysis of acetylated α-tubulin immunoreactive bands in all the experimental conditions shown in the left panel. **(C)** Left panel: representative western blot of cells surface biotinylated Nav1.5 (biot-Nav1.5) and Nav1.5 in cell lysates (Nav1.5) in the absence (–) or presence (+) of colchicine (colchi) in LMNA WT and LMNA Q517X-expressing HL-1 cells; right panel: normalized densitometric analysis of biot-Nav1.5 immunoreactive bands in all the experimental conditions shown in the left panel. The data are means of 3 independent experiments *p < 0.01, **p < 0.001, one-way Anova.

An FDA-approved alkaloid, colchicine, inhibits tubulin polymerization in heart (Deftereos et al., 2013) and depolymerizes the tubulin network of LMNA Q517X-expressing cardiomyocytes to the same extent of control cells (Figure 6A, LMNA Q517X + colchi).

Concomitantly, colchicine treatment reduced the levels of acetylated α-tubulin (Figure 6B) and increased the cell surface expression of Nav1.5 in LMNA Q517X-expressing cardiomyocytes (Figure 6C) toward the levels of those found in LMNA WT-expressing cardiomyocytes.

**FIGURE 7:**
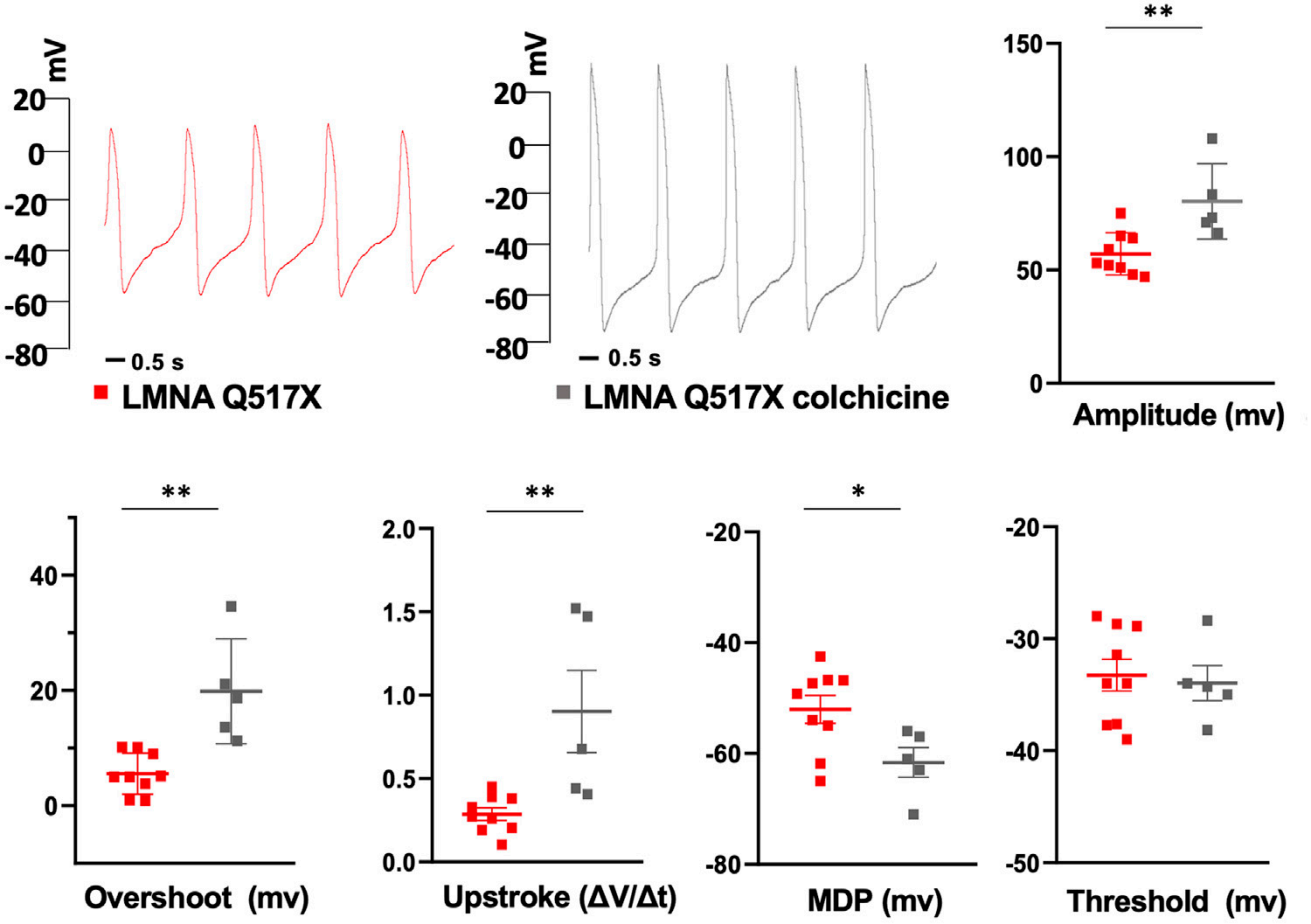
Representative AP recordings by whole-cell patch clamp in current clamp configuration in untreated (red traces) and colchicine-treated LMNA Q517X-expressing HL-1 cells (gray traces) and scatter plots for the analysis of AP parameters in untreated (red squares, *N* = 9) and colchicine-treated LMNA Q517X-expressing HL-1 cells (gray squares, *N* = 5). *p < 0.01, **p < 0.001, Student’s t test for unpaired data.

Interestingly, Figure 7 shows that the treatment of HL-1 cardiomyocytes expressing LMNA Q517X with colchicine leads to a WT-like shape of AP when compared with untreated cardiac cells (red traces for LMNA Q517X, gray traces for LMNA Q517X + colchicine). Moreover, colchicine treatment induced a significant recovery of all AP’s parameters measured in LMNA Q517X cardiomyocytes toward those found in LMNA WT-expressing cardiomyocytes. Specifically, as shown in the scatter plots (Figure 7, scatter plots) AP amplitude (amplitude in mV: red squares 57.11 ± 3.11, vs gray squares 80.32 ± 7.45), AP overshoot (overshoots in mV: red squares 5.58 ± 1.17, vs gray squares 19.88 ± 4.07), maximum upstroke velocity (upstroke in mV/ms: red squares 0.28 ± 0.03, *vs* gray squares 0.90 ± 0.24), and the maximum diastolic potential (MDP in mV: red squares −52.03 ± 2.50, *vs* gray squares -61.6 ± 2.675) were all significantly increased in LMNA Q517X-treated cardiomyocytes compared with their controls. No significant changes were observed in APs’ threshold between the two experimental conditions (thresholds in mV: red squares −33.25 ± 1.41, *vs* gray squares −33.96 ± 1.57).

**FIGURE 8:**
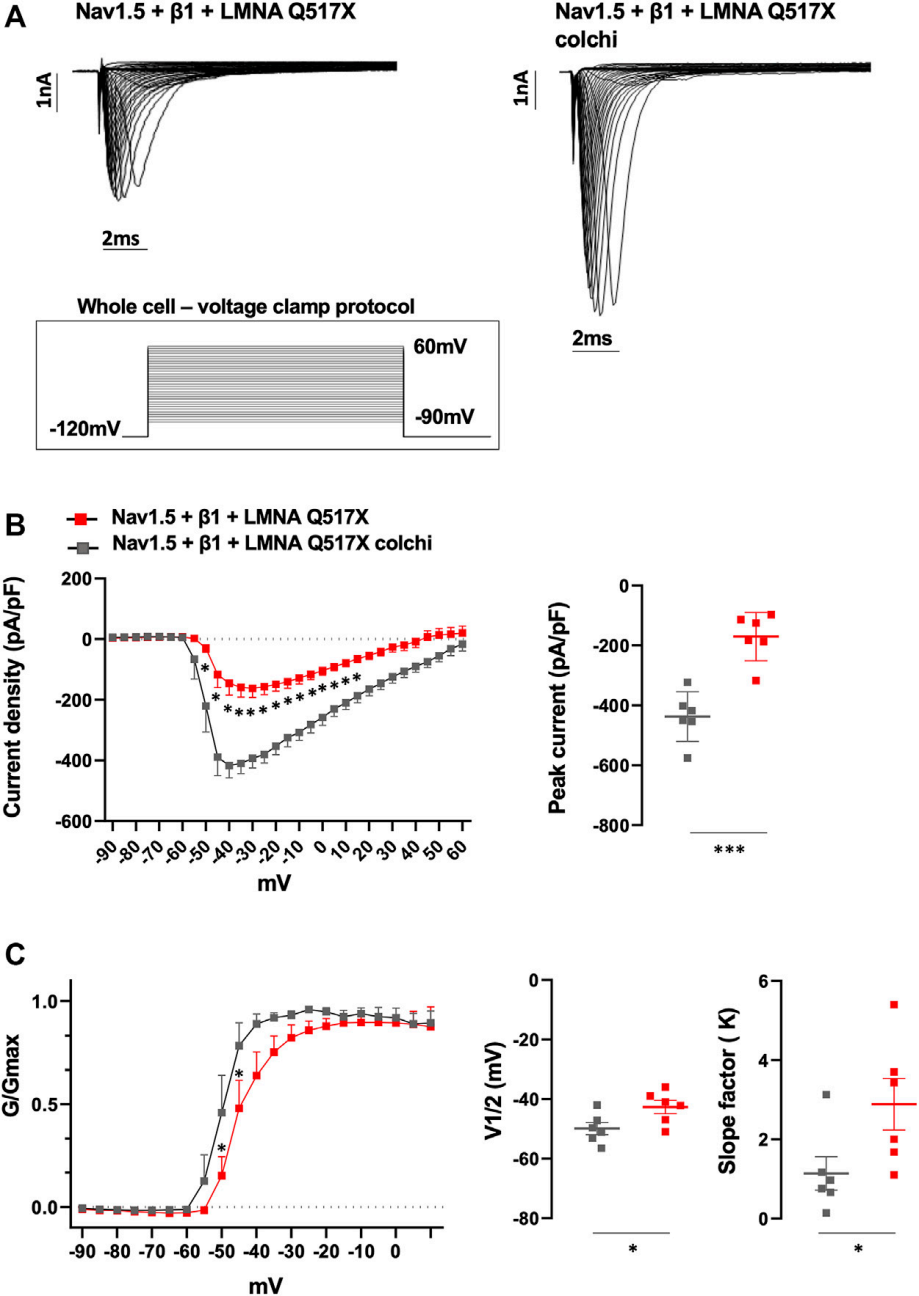
**(A)** Representative traces of inward Na^+^ currents evoked in untreated (Nav1.5 + β1 + LMNA Q517X) or colchicine-treated LMNA Q517X-expressing HEK293 cells (Nav1.5 + β1 + LMNA Q517X colchi). **(B)** Left panel: current density (pA/pF)–voltage relationships (I–V curve) of the Na^+^ currents recorded in untreated (red trace, *N* = 6), and colchicine-treated LMNA Q517X-expressing HEK293 cells (gray trace, *N* = 6); *p*-value < 0,0001 from −50 mV to −10 mV; *p*-value< 0.001 at −5 mV and at 0 mV; *p*-value < 0.01 at 5 mV and *p*-value < 0,05 at 10 mV and 15 mV, two-way Anova. Right panel: scatter plot of the maximum peak sodium currents (pA/pF) in untreated (red squares) and in colchicine-treated LMNA Q517X-expressing HEK293 cells (gray squares). **(C)** Left panel: conductance voltage (G/Gmax) relationships of Na^+^ currents (G–V curve) obtained for untreated (red trace, *N* = 6), and colchicine-treated LMNA Q517X-expressing HEK293 cells (gray trace, *N* = 6); *p*-value < 0.001 at −50 mV and at −45 mV, two-way Anova. Right panel: scatter plot of the half-maximal conductance (V1⁄2) and slope factors (K) in untreated (red squares) and in colchicine-treated LMNA Q517X-expressing HEK293 cells (gray squares); *p < 0.01; **p < 0.001, Student’s t test for unpaired data.

Similarly, colchicine treatment reversed the Nav1.5 biophysical parameters measured in LMNA Q517X-expressing HEK293 cells toward those measured in LMNA WT-expressing HEK293 cells. As shown in Figure 8A (representative current traces) and 8B (current density plots: red line for Nav1.5 + β1 + LMNA Q517X, gray line for Nav1.5 + β1 + LMNA Q517X + colchicine), Na^+^ currents recorded in HEK293 cells expressing LMNA Q517X significantly increased upon colchicine treatment. The peak current of Na^+^ returned to that observed in LMNA WT-expressing-HEK293 cells (Figure 8B, peak current in pA/pF: red squares −170.5 ± 32,942 *vs* gray squares −437,166 ± 33,843). Upon colchicine treatment the steady-state activation curve of Nav1.5 in LMNA Q517X-expressing cells returned to that observed in LMNA WT-expressing cells (Figure 8C, G/Gmax: red line for Nav1.5 + β1 + LMNA Q517X, gray line for Nav1.5 + β1 + LMNA Q517X + colchicine). Finally, upon colchicine treatment both V1/2 and slope factor values measured in LMNA Q517X-expressing HEK293 returned to those observed in LMNA WT-expressing cells (Figure 8C; V1/2 in mV: red squares −42.68 ± 2.235 *vs* gray squares −49.93 ± 2.034; slope factor in mV: red squares 2.886 ± 0.650 *vs* gray squares 1.141 ± 0.422).

## Discussion

In this work, we found that the pathogenetic LMNA Q517X variant, through a modulation of the microtubule meshwork state, impairs Nav1.5 expression and function at the plasma membrane in the murine atrial cell line HL-1, thus proving new insights into both the way how LMNA may regulate cardiac function in physiopathological conditions and the possible therapeutic approaches in the field of cardiac laminopathies.

As a consequence of the reduced Nav1.5 activity at the plasma membrane, APs have reduced amplitude, overshoot, upstroke velocity, and increased diastolic potential in LMNA Q517X-expressing cardiomyocytes compared with their controls. These AP features may decrease conduction velocity in atrial cardiomyocytes and autorhythmic cells inducing atrial fibrillation, sick sinus syndrome, and AV-block, all clinical findings of the index family carrying LMNA Q517X mutant (Figure 1A, results). Atrial fibrillation is one of the most common causes for cardiac function deterioration in the setting of DCM (Middlekauff et al., 1991; Mamas et al., 2009), which also occurred in LMNA Q517X carriers. Accordingly, loss-of-function mutations in Nav1.5 encoding gene, *scn5a*, have been found associated with atrial fibrillation and conduction defects (Olson et al., 2005; Darbar et al., 2008). Of note, loss of function in the Nav1.5 channel can be also associated with DCM and ventricular arrhythmias, regardless of whether the atrial fibrillation occurred, suggesting that reduction in the inward Na^+^ current may affect directly electrical properties and fates of ventricular cardiomyocytes (McNair et al., 2004). Indeed, we cannot exclude that LMNA Q517X variant expressed in the whole heart of carriers may affect directly ventricular function.

The clinical history of the index family carrying LMNA Q517X mutant, however, puts the permanent atrial fibrillation and conduction defects showed at the onset of the cardiomyopathy as key events for the following heart failure.

We found that LMNA Q517X expression induced both microtubules hyper-polymerization and Nav1.5 downregulation at the plasma membrane in atrial HL-1 cardiomyocytes and significant reduction in the peak I_Na_ amplitude in Nav1.5-expressing HEK293 cells. These results are in agreement with the findings of Casini et al., showing that treatment with the anticancer drug Taxol, which polymerizes tubulin, reduces sarcolemmal Nav1.5 in neonatal cardiomyocytes, and I_Na_ amplitude in Na1.5-expressing HEK293 cells of about two-fold (Casini et al., 2010).

The density of the microtubule alters microtubule interactome not only affecting channel delivery/turnover (Steele and Fedida, 2014), but also E-C coupling, myofilament contractility, and proteostasis (Chinnakkannu et al., 2010), thus accounting also for structural cardiac abnormality.

Interestingly, the increase in α tubulin density is paralleled by a significant increase in the acetylation level of α tubulin at 40 lisine (K40) in the amino acidic sequence on α tubulin (Figure 3C), as expected by the known higher affinity of the tubulin acetyltransferase (aTAT1) for polymerized tubulin (Janke and Chloë Bulinski, 2011). It was demonstrated that K40 acetylation weakens lateral interactions between protofilaments, thus softening the microtubules (Portran et al., 2017). As microtubules in living cells are frequently exposed to mechanical forces, an acetylation-induced increase in their flexibility would allow microtubules to better resist mechanical stress consequently making acetylated microtubules more long-lived. At the same time, longer-lived microtubules are still more likely to experience mechanical stress, thus further accumulating acetylation marks, which could reflect the notion that acetylation is a marker of microtubule age.

Indeed, it can be hypothesized that LMNA Q517X mutant impairing the mechanical nuclear–cytoskeletal coupling induces an adaptive cellular response such as microtubules polymerization and acetylation mimicking aged microtubules, with consequences on dis-regulated trafficking of Nav1.5 channel at the cell surface and the creation of pro-arrhythmic substrate.

The increasing in the α-tubulin acetylation may also account for the upregulation of AKT pathway we also found in LMNA Q517X-expressing cardiomyocytes. Interestingly, Giustignani et al. found that tubulin acetylation recruits the chaperon protein Hsp90 to microtubules stimulating the signaling pathways of its client kinase AKT (Giustiniani et al., 2009).

Of note, microtubules polymerization and stabilization obtained by either Taxol or Discodermolide two known chemotherapies, significantly increased ERK1/2 phosphorylation in different cell lines (Seidman et al., 2001; Klein et al., 2005). Accordingly, we cannot exclude that the activation of ERK1/2 pathway we found in LMNA Q517X-expressing cardiomyocytes may be a downstream process to microtubules hyper-polymerization we found associated with this LMNA variant.

The altered tubulin state has already been found associated with some LMNA pathogenic variants. Instable microtubule network with a decrease in the acetylation of α tubulin triggered abnormal CX43 localization and consequent electrical communication between cardiomyocytes in Lmna^H222P/H222P^ and Lmna^N195K/N195K^ mice (Macquart et al., 2019; Borin et al., 2020). Accordingly, stabilization of microtubules using paclitaxel, a microtubule-stabilization agent commonly used in chemotherapy, repositioned CX43 in cardiomyocytes of Lmna^N195K/N195K^ mice and improved intraventricular conduction defects in these mice (Macquart et al., 2019). Recently, instable microtubules meshwork was also found in cardiomyocytes expressing E161K and D192G pathogenic LMNA variants with again a displacement of CX43 (Borin et al., 2020), demonstrating a different pathogenic mechanism by which LMNA may displace a sarcolemmal protein through the microtubule network thus impairing heart electrical physiology.

To the best of our knowledge, we reported for the first time that an LMNA pathogenic variant is associated with an excess of microtubule polymerization and stabilization, which impairs Nav1.5 sarcolemmal expression and biophysics generating abnormalities in AP generation and propagation in cardiomyocytes.

Interestingly, we showed that a well-known FDA-approved alkaloid, colchicine, which depolymerizes the tubulin meshwork, reverted the altered AP properties of LMNA Q517X-expressing cardiomyocytes recapitulating those of the LMNA WT-expressing cardiomyocytes.

Similarly, when colchicine was tested on HEK293 cells expressing Nav1.5 and LMNA Q517X, I_Na_ currents and kinetics returned to those measured in control cells. The effect of colchicine non only provided evidence that the altered state of tubulin we observed is actually the key event in the electrical features of mutant expressing cardiomyocytes but also suggested a therapeutic intervention for this LMNA cardiomyopathy.

Interestingly, *in vivo* colchicine treatment significantly reduced microtubule density in cardiomyocytes of different rat models (Prins et al., 2017; Scarborough et al., 2021). Moreover, in the last 15 years colchicine was introduced in the field of cardiology for the treatment and prevention of different cardiovascular diseases (Fiolet et al., 2021), thus suggesting colchicine as a possible ready-to-use treatment for LMNA Q517X carriers.

In conclusion, our results confirm that LMNA protein at the nuclear envelope controls a chain of events involved not only in the mechanical but also in the electrical signaling transfer from the nucleus to the cell membrane in cardiomyocytes. Moreover, our findings suggest that every LMNA mutant may act with different pathogenic mechanisms in generating a common clinical phenotype in heart such as arrythmias and conduction defects. Indeed, it is very important to characterize each LMNA pathogenic variant in cardiomyocytes to gain insights on both the complexity of the cardiac cell biology, the mechanisms leading to cardiac dysfunction in laminopathies and the possible therapeutic approaches.

## Data Availability

The raw data supporting the conclusion of this article will be made available by the authors, without undue reservation.
