# Supplementary material for: Role of Nuclear Lamin A/C in the Regulation of Nav1.5 Channel and Microtubules: Lesson From the Pathogenic Lamin A/C Variant Q517X
Source: Front Cell Dev Biol. 2022 Jun 29;10:918760. doi: 10.3389/fcell.2022.918760 (PMC9277463; doi:10.3389/fcell.2022.918760)
Supplement: Supplementary file 1 [file DataSheet1.PDF]

A

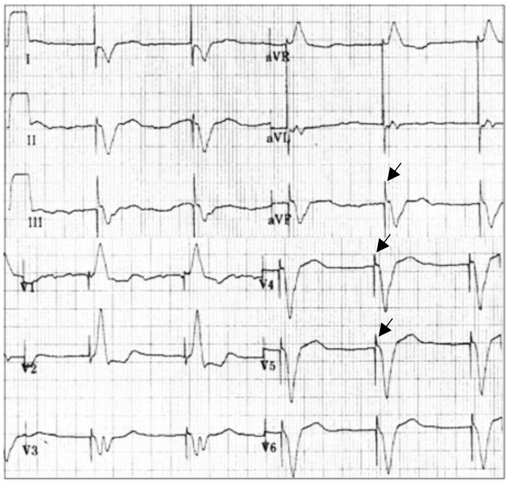

B

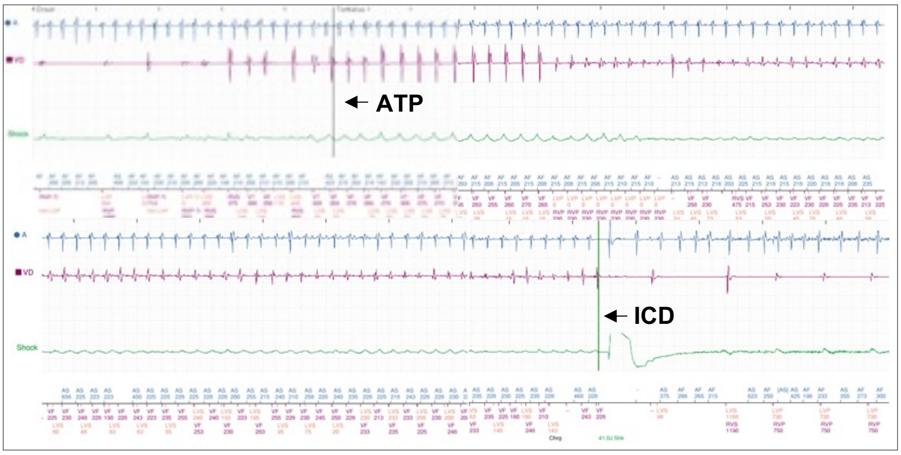

Supplementary Figure 1: A) 12-lead ECG from index patient showing atrial fibrillation and ventricular rhythm induced by a pacemaker with VVI pacing mode. Arrows indicate pacemaker spikes. B) Typical VF episode recorded in the index patient and stopped by internal ICD shock after ineffective ATP. ATP, anti-tachycardia pacing; ECG, electrocardiogram; ICD, implantable cardioverter-defibrillator;

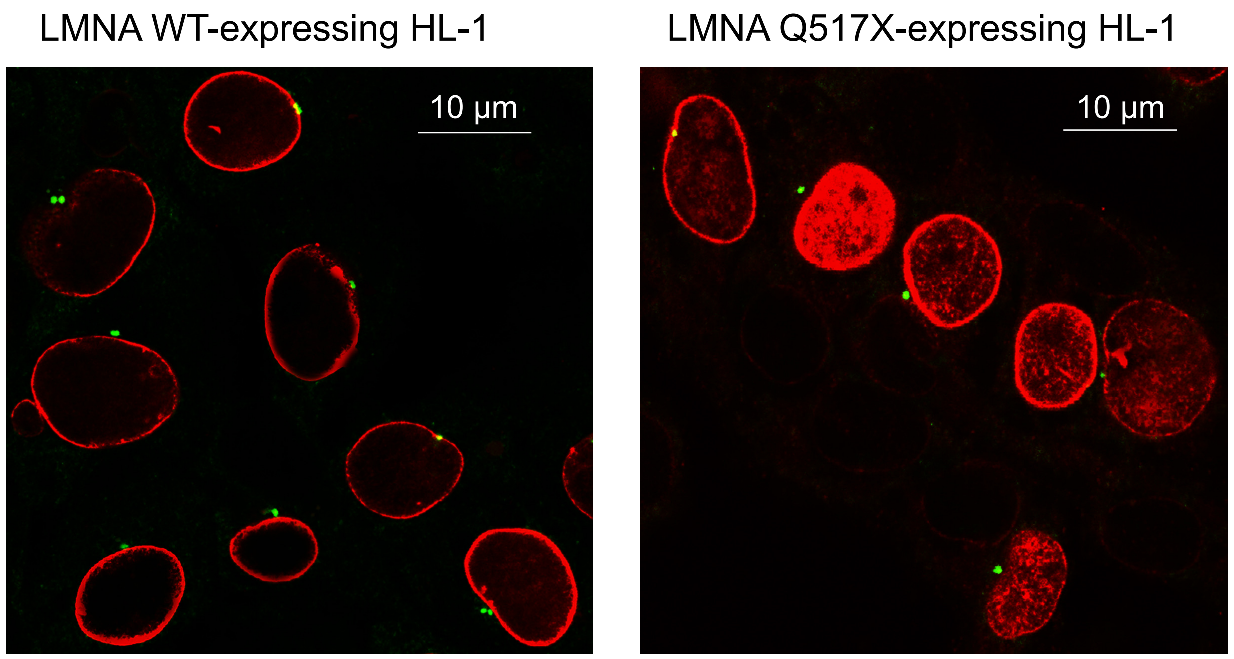

Supplementary Figure 2: Representative immunofluorescence confocal images of HL-1 cells expressing either LMNA WT or LMNA Q517X (red signal) labelled with the antibody anti- γ tubulin which marks the microtubule-organizing-center MTOC (green signal).

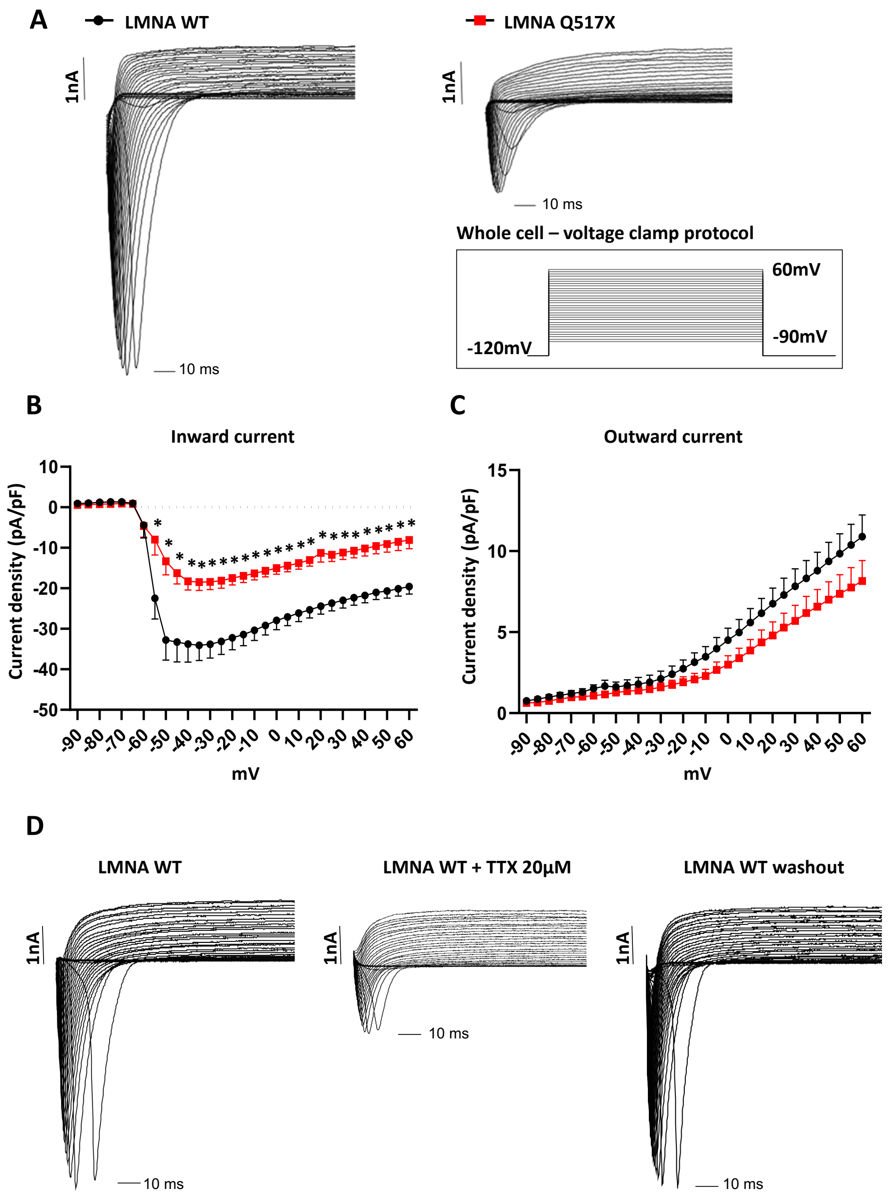

Supplementary Figure 3: A) representative traces of currents recorded, using a voltage protocol consisting of a holding potential of −120 mV followed by depolarizing steps from −90 to +60 mV (with increments of 5 mV), in HL-1 cardiomyocytes stably expressing either LMNA Q517X (n=11) or LMNA WT (n=14). B) I-V plot of the inward current measured as the peak inward current evoked at each depolarizing step. The inward currents appear significantly smaller in LMNA Q517X expressing HL-1 cells compared with their control cells (with a peak current of -34,09 ± 3,71 pA/pF, in cells expressing LMNA WT and a peak current of -18,47 ± 2,02 pA/pF in cells expressing LMNA Q517X, A-nova, p-value: 0,0002). C) I-V plot of the outward current measured as the outward current evoked at the end of each depolarizing step. No significative differences were detected between the outward current recorded in HL-1 cells expressing either LMNA Q517X or LMNA WT (with an evoked outward current at 60 mv of 10,89 ± 1,34 pA/pF in cells expressing LMNA WT and, at the same voltage, an outward current of 8,15 ± 1,2 pA/pF in cells expressing LMNA Q517X, A-nova, p-value: 0,2). D) Representative traces of currents recorded in HL-1 cardiomyocytes stably expressing LMNA WT (LMNA WT), after application of 20 µM tetrodotoxin in the extracellular solution (LMNA WT+ 20µM TTX) and after tetrodotoxin washout (LMNA WT washout).

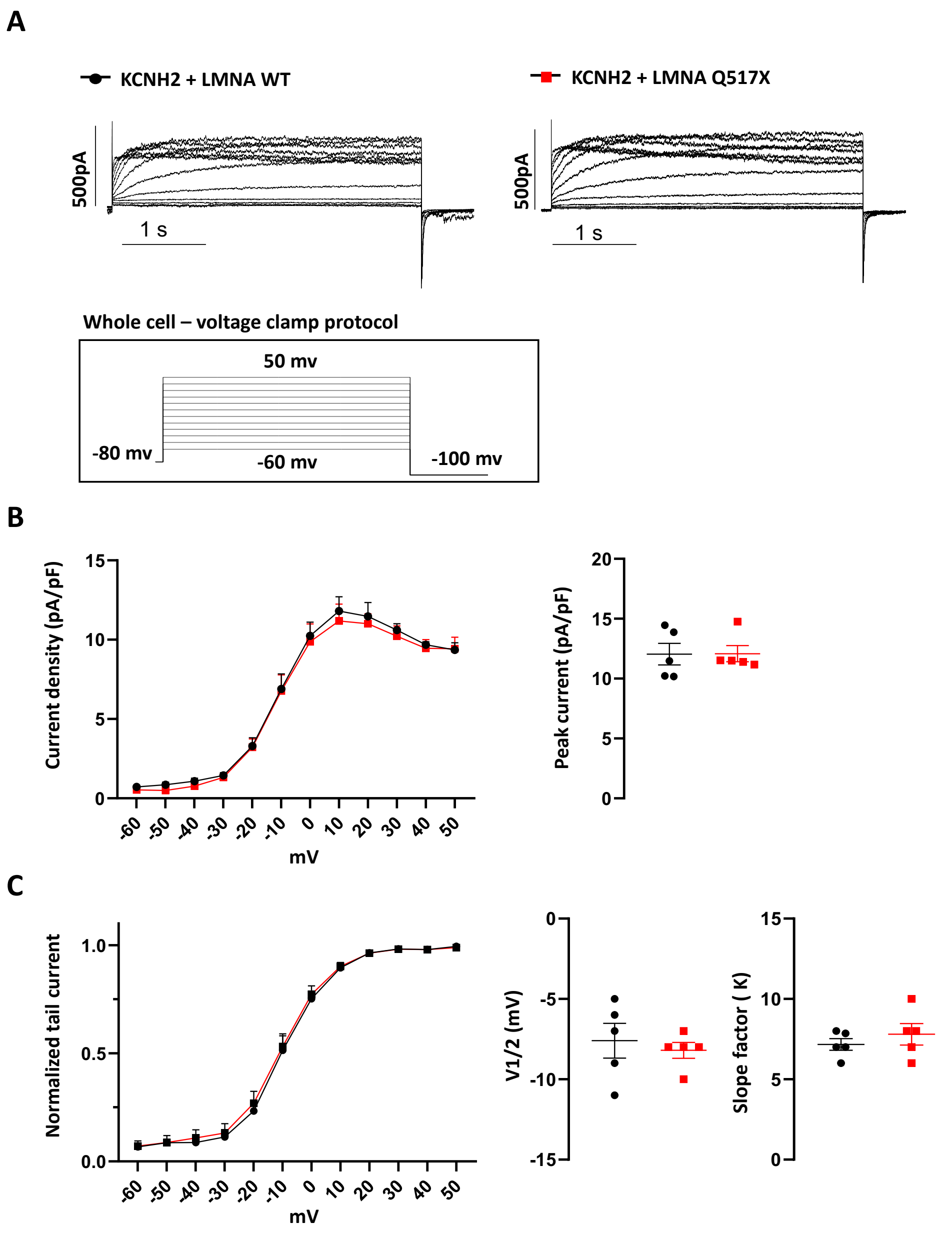

Supplementary Figure 4: A) representative traces of the K^+ currents recorded, using a voltage protocol consisting of a holding potential of −80 mV followed by 4s depolarizing steps from −60 to +50 mV (with increments of 10 mV), in HEK293 cells transiently co-transfected with KCNH2 and either LMNA Q517X (n=5) or LMNA WT (n=5) expression plasmids. B) I-V plot of the evoked outward K^+ current in cells described above. The K^+ current, measured at the end of the depolarizing steps, increased at each further depolarizing step until reached a similar maximum outward current amplitude in both experimental conditions at approximately 10 mv (with a peak current of 12,03 ± 0,9 pA/pF, in cells expressing LMNA WT and a peak current of 12,07 ± 0,67 pA/pF in cells expressing LMNA Q517X, t-test, p-value: 0,9749). After reaching the peak, both I-V curves quickly decreased at more positive potentials because of the voltage-dependent KCNH2 channel inactivation. C) Activation curves obtained fitting to a Boltzmann relationship the K^+ tail current normalized to the maximum tail current amplitude. The activation curves obtained confirmed a comparable channel kinetics in both experimental conditions (with a half-maximum activation voltage (V1/2) of −7,6 ± 1,07 mV and a slope factor (κ) of 7,1 ± 0,35 for HEK293 cells expressing LMNA WT and a half-

maximum activation voltage (V1/2) of −8,2 ± 0,49 mV and a slope factor (κ) of 7,8 ± 0,66 for HEK293 cells expressing LMNA Q517X; t-test, V1/2 p-value: 0,62, K p-value: 0,42). Data were given as mean ± SEM.
